# Association between Initial Serum Total Bilirubin and Clinical Outcome in Myocardial Infarction with Non-Obstructive Coronary Arteries

**DOI:** 10.7150/ijms.70833

**Published:** 2022-05-27

**Authors:** Guoqing Yin, Lu Liu, Abdul-Quddus Mohammed, Rong Jiang, Fuad A. Abdu, Wenliang Che

**Affiliations:** 1 Department of Cardiology, Shanghai Tenth People's Hospital, Tongji University School of Medicine; Shanghai, China; 2 Department of Cardiology, Shanghai Tenth People's Hospital Chongming branch, Shanghai, China

**Keywords:** MINOCA, Total bilirubin, clinical outcomes, Predictors

## Abstract

**Background:** Liver function parameters, particularly serum total bilirubin (TB), are closely associated with cardiovascular diseases. However, the impact of serum TB among patients with myocardial infarction and non-obstructive coronary (MINOCA) remains unknown. Our study investigated the relationship between serum TB at admission and long-term adverse clinical outcomes in MINOCA patients.

**Methods:** A total of 273 consecutive MINOCA patients were categorized into low and high serum TB groups based on the optimal cut-off of 0.9 mg/dl. The primary endpoint was major adverse cardiovascular events (MACE), including cardiac death, non-fatal MI, heart failure, and angina rehospitalization. Receiver-operating characteristic, Cox regression, and Kaplan-Meier analyses were used to evaluate the association of high serum TB with cardiovascular outcomes.

**Results:** High serum TB was found in 68 (24.9%) patients. The incidence of MACE was higher in the high TB group than in the low TB group after a median follow-up of 28 months (30.9 vs. 17.1%, *P*=0.015). The Kaplan-Meier curve analysis also indicated that patients in the high TB group had a higher risk of developing MACE (log-rank *P*=0.023). Cox regression analysis showed that high serum TB (>0.9mg/dl) significantly correlated with increased MACE risk (HR=1.90, 95%CI: 1.12-3.22, *P*=0.018). After adjusting for numerous clinical variables, the high serum TB remained significantly associated with an increased risk of MACE (HR=2.04, 95%CI: 1.05-3.94, *P*=0.034).

**Conclusion:** High initial serum TB (>0.9mg/dl) is a robust predictor of poor clinical outcomes among MINOCA patients. In clinical settings, assessing serum TB at admission may help identify high-risk patients presenting with MINOCA.

## Introduction

Myocardial infarction and non-obstructive coronary arteries (MINOCA) refers to a special category of acute myocardial infarction (AMI) where the stenosis of epicardial arteries is less than 50% 1, 2, and accounts for nearly 1% to 15% of total AMI cases 3-5. Earlier studies have shown that MINOCA is an unfavorable condition, and these patients are still at an increased risk of major adverse cardiovascular events (MACE) after a short or long-term follow-up period 6-8. Although MINOCA is relatively frequent and is associated with poor prognosis, the current clinical management in this patient group remains controversial, mainly due to the uncertain etiology, risk factors, and lack of evidence-based clinical studies. Therefore, it is potentially important to identify new predictive biomarkers to improve the clinical outcomes and provide individualized treatment options and guidance in the clinical work of MINOCA patients.

Elevated liver function parameters such as alanine aminotransferase, aspartate aminotransferase, and alkaline phosphatase are frequently detected in AMI patients, and these abnormal biochemical parameters have been shown to be related to worse outcomes 9. Serum total bilirubin (TB), an end product of heme metabolism, is another valuable serum biomarker for assessing adverse outcomes in coronary artery disease 10, 11. However, the prognostic value of serum TB is controversial; some studies have shown that serum TB has a preventive impact on diabetes mellitus, hypertension, and metabolic syndrome 12-14. In contrast, this impact is different in acute clinical conditions such as AMI, which showed that elevated serum TB is positively associated with worse cardiovascular outcomes in patients with AMI 15-18. However, there have been no relevant studies evaluating the impact of liver enzymes or serum TB on clinical outcomes among the MINOCA population.

Thus, the present study sought to investigate the association between initial serum TB and unfavorable cardiovascular outcomes in MINOCA patients during long-term follow-up.

## Methods

### Study population and data collection

We enrolled all AMI patients who were admitted to the cardiology department of Shanghai Tenth People's Hospital and who underwent coronary angiography from 2015 to 2019. Patients were classified as MINOCA if they met the diagnostic criteria for AMI outlined in the 4th universal definition of myocardial infarction (MI)^2^, and their epicardial coronaries revealed no stenosis > 50% during coronary angiography. The exclusion criteria of this study were as follows: 1) age < 18; 2) patients with type 3-5 myocardial infarction; 3) patients receiving thrombolytic therapy before coronary angiography examination; 4) patients with typical myocarditis, Takotsubo syndrome, or pulmonary embolism; 5) patients with no data of serum TB or TB >2.0 mg/dl; and 6) patients lost to follow-up. Eventually, 273 MINOCA patients were included in the final analysis of the present study.

Baseline characteristics such as demographic information, past medical history, laboratory test, electrocardiogram, and echocardiography results were obtained from their medical records. Fasting venous blood samples were obtained within 24 hours of admission to assess biochemical analysis. TB was measured using Bilirubin Total Gen.3 reagent (Roche Diagnostics GmbH, Mannheim, Germany), and standard procedures were used to evaluate the other biochemical parameters. The normal range of TB was 0.2 to 1.2 mg/dl in our study. The optimal cut-off of 0.9mg/dl was obtained from receiver operating characteristic curve (ROC) analysis based on MACE prediction and was used to distinguish the groups in our study.

The current study was approved by the Ethics Committee of Shanghai Tenth People's Hospital and was carried out in accordance with the Helsinki Declaration. Each participant in this study signed a written informed consent form.

### Follow-up and endpoints

The median follow-up period of this study was 28 months. The follow-up data were collected from the health record of patients, outpatient care, telephone calls, and hospital records. The endpoint of the present study was MACE, which was defined as cardiac death, non-fatal MI, heart failure, and angina rehospitalization. Cardiac death was defined as death caused by heart failure, arrhythmia, or sudden death without any other explanation. MI was diagnosed according to the 4th universal definition 2. Heart failure was diagnosed based on the current guidelines 19. Angina rehospitalization was defined as the presence of objective evidence of ischemia (electrocardiographic changes during spontaneous pain episodes at rest) requiring rehospitalization. The endpoints were confirmed by two qualified cardiologists.

### Statistical analysis

MINOCA patients were categorized into two groups based on the optimal cut-off of 0.9 mg/dl, namely, high TB group (>0.90 mg/dl) and low TB group (≤0.90 mg/dl). The optimal cut-off value was established by ROC analysis based on MACE prediction, and the area under the curve (AUC) was estimated. Continuous variables were represented as mean ± standard deviation, whereas categorical variables were displayed as numbers with percentages. For continuous variables, the independent sample t-test was used, and for categorical variables, the chi-square test or Fisher's exact test was used. Kaplan-Meier method was used to create the cumulative survival curve for MACE, and log-rank tests were used to test for differences. Cox regression analysis, both univariable and multivariable, was used to examine the association between serum TB and clinical outcomes. The risk of MACE was adjusted for age and sex (Model 1) and multivariable adjustment, which includes age, sex, body mass index (BMI), diabetes, hypertension, hyperlipemia, atrial fibrillation, heart failure, and left ventricular ejection fraction (Model 2). The hazard ratio (HR) with a 95% confidence interval (CI) was calculated. A two-tailed *P*<0.05 was considered statistically significant. SPSS software (version 24.0, Chicago, IL, USA) was used to analyze the data of this study, and GraphPad Prim software (version 8.0.1; Inc, USA) was used to create the graphics.

## Results

**Figure 1:**
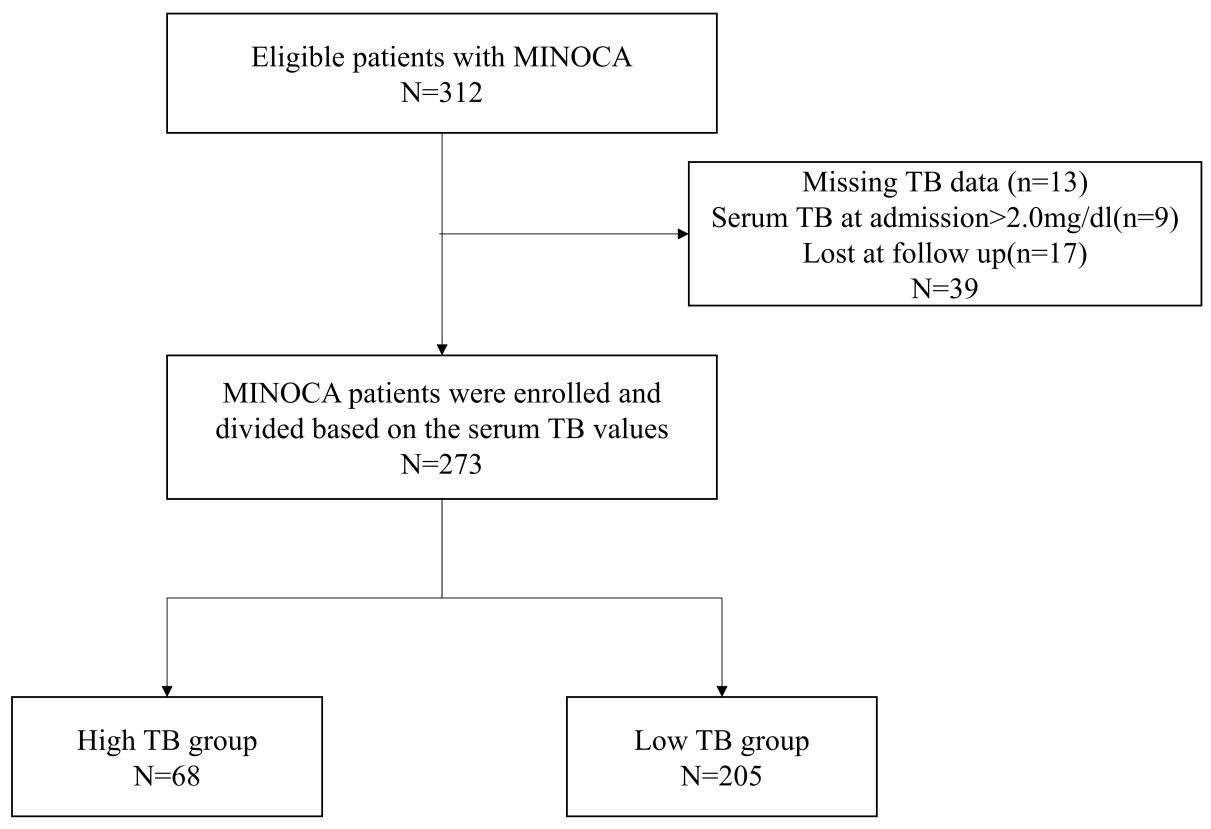
Flow chart of the study.

A total of 312 patients diagnosed with MINOCA were enrolled consecutively, of which 39 were excluded from the study due to missing serum TB data (n=13), loss to follow-up (n=17), and serum TB >2.0 mg/dl at admission (n=9). Finally, 273 MINOCA patients were divided in two groups based on the value of serum TB at admission (high TB group: serum TB>0.9mg/dl, n=68; low TB group: serum TB≤0.9mg/dl, n=205) (Figure 1).

### Baseline characteristics

Baseline characteristics and laboratory findings of all MINOCA patients in the high and low TB groups are shown in Table 1. Patients in the low TB group had higher LVEF values than patients in the high TB group (56.31±10.25 vs. 52.08±12.47, *P*=0.003). In addition, the triglyceride levels of patients in the low TB group were also significantly higher than those in the high TB group (1.62±1.18 vs. 1.23±0.67, *P*=0.029). Age, sex, BMI, history of hypertension, diabetes, dyslipidemia, atrial fibrillation, heart failure, admission characteristics, and laboratory findings did not differ significantly between the two groups (all *P*>0.05).

### Clinical outcomes stratified by a TB value of 0.9mg/dl

**Figure 2:**
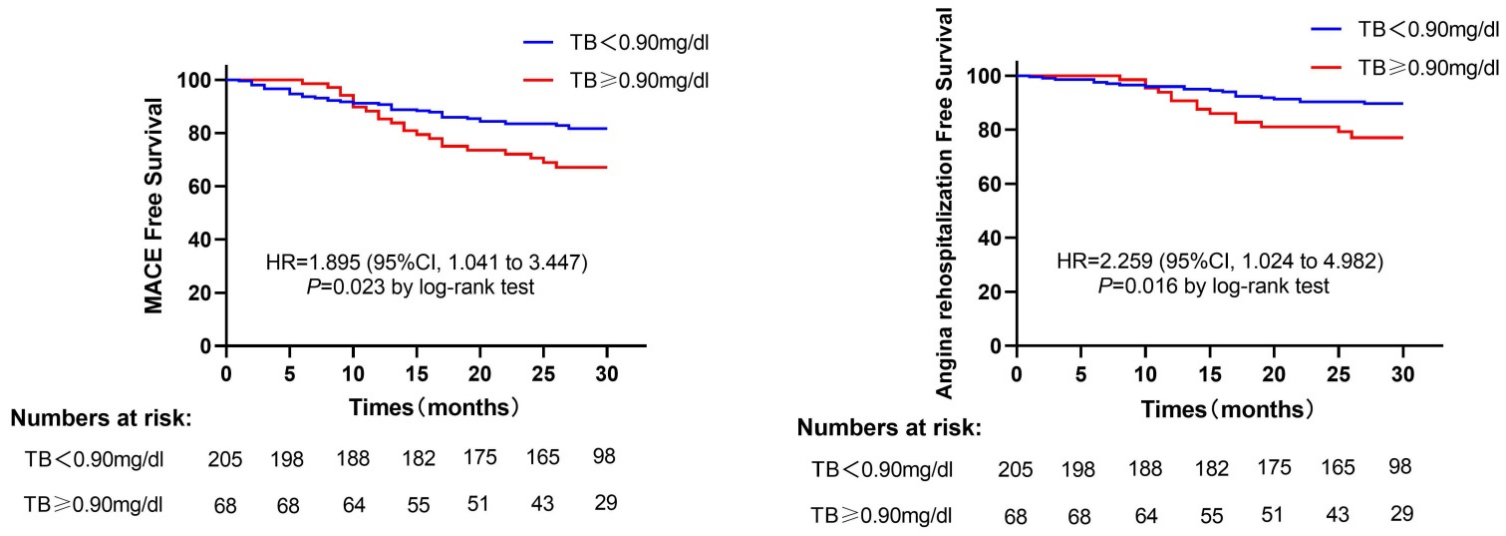
(A) Kaplan-Meier survival curves for MACE in MINOCA patients in the high TB group versus low TB group. (B) Kaplan-Meier survival curves for angina rehospitalization in MINOCA patients in high TB group versus low TB group. Abbreviations: MACE, major adverse cardiovascular events; TB, total bilirubin; HR, hazard ratio; CI, confidence interval.

A total of 56 patients suffered MACE after a median follow-up of 28 months (17 cardiac deaths, 2 non-fatal MI, 3 heart failures, 34 angina rehospitalizations), 35 patients in the low TB group patients, and 21 patients in the high TB group patients. The incidence of MACE was significantly higher in the high TB group than those in the low TB group (30.9% vs. 17.1%, *P*=0.015). The angina rehospitalization was also higher in the high TB group compared with the low TB group (20.6% vs. 9.8%, *P*=0.015) (Table 2). Furthermore, the Kaplan-Meier curve showed that patients with TB>0.9mg/dl have an increased risk of developing MACE (log-rank *P*=0.023) (Figure 2A). Likewise, the same results were reported when only angina rehospitalization was analyzed (log-rank *P*=0.016) (Figure 2B).

### The association between initial serum TB and long-term clinical outcomes

The association between initial serum TB and clinical outcomes is displayed in Table 3. Univariate Cox regression analysis showed that high serum TB (>0.9mg/dl) was associated with a higher risk of MACE (HR=1.90, 95%CI: 1.12-3.22, *P*=0.018). On multivariate Cox regression analysis, after adjusting for age and sex, patients in the TB >0.9mg/dl group also had a significantly higher risk of MACE (HR=1.83, 95%CI: 1.08-3.12, *P*=0.026). After adjusting for multiple clinical variables (model 2), Cox regression analysis showed that the high serum TB was still independently associated with a higher risk of developing MACE after long-term follow-up (HR=2.04, 95%CI: 1.05-3.94, *P*=0.034). Furthermore, the value of serum TB was positively associated with an elevated risk of MACE (per 1SD of TB increase, HR=1.43, 95% CI: 0.07-0.92, *P*=0.016), indicating that high serum TB (TB>0.9mg/dl) was a robust risk predictor of developing MACE among patients with MINOCA (Table 3).

**Figure 3:**
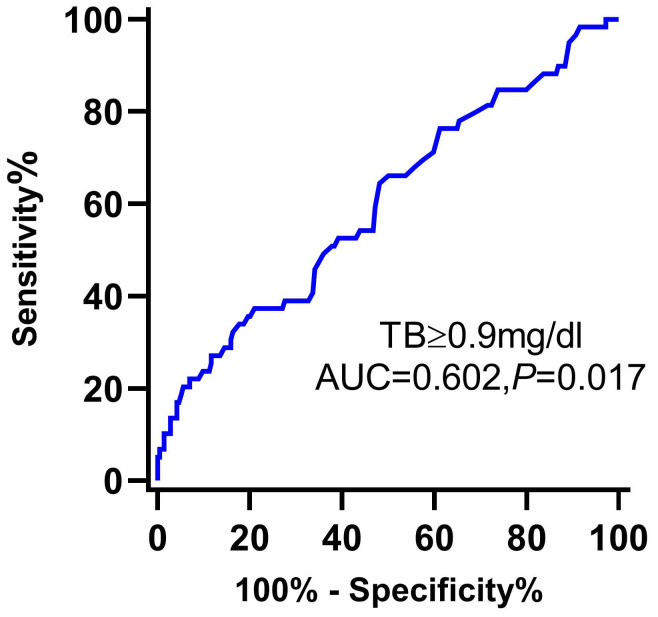
Receiver operating characteristic curve of TB for the prediction of the long-term MACE of MINOCA patients. Abbreviations: TB, total bilirubin; AUC, area under the curve; CI, confidence interval.

The ROC curve of serum TB to predict the long-term MACE of MINOCA is shown in Figure 3.

## Discussion

This is the first study to investigate the impact of serum TB on MACE in patients suffering from MINOCA. The novel findings of the present study were: 1) MINOCA patients with high serum TB (>0.9mg/dl) had a significantly higher risk of developing MACE than those with lower serum TB (≤0.9mg/dl); 2) initial serum TB>0.9mg/dl was an independent predictor of long-term MACE of MINOCA population. We demonstrated a positive association between initial serum TB and long-term clinical outcomes among MINOCA patients, highlighting its prognostic value in the risk stratification in the current treatment of MINOCA patients.

With coronary angiography widely used, MINOCA, a special type of AMI, has become increasingly recognized in current clinical practice 1, 4. MINOCA is common in female and young patients and often presents with non-ST-elevation myocardial infarction 3, 20. Several studies have revealed that patients with MINOCA had slightly better clinical outcomes than AMI patients with obstructive coronary arteries 6, 21. However, this finding is not consistent across all studies; in a large retrospective research involving 6063 MINOCA patients, the all-cause mortality rate at one year and three years was 10.94% and 16.18%, respectively 22. Another recent multicenter observational cohort research indicated that one of every five MINOCA patients suffers MACE after the follow-up of one year 23. According to the TOTAL-AMI study, 21.5% of MINOCA patients developed MACE during a median follow-up of 3.8 years 6. In line with previous studies, the incidence of MACE was 20.5% after a median follow-up of 28 months, highlighting the importance of physicians paying special attention to this population. Despite the high prevalence rate and poor clinical outcomes, there is a scarcity of information on the risk factors for the poor clinical outcomes of MINOCA. As a result, particular attention needs to be given to MINOCA to identify potential risk factors and stratify these patients timely, thereby improving MINOCA management and prognosis.

Bilirubin, previously considered a useless by-product of heme metabolism, has recently been found to be a valuable antioxidant with cytoprotective effects 24, 25 and has been demonstrated to have anti-inflammatory effects 26. Given the anti-inflammatory and antioxidant effects of serum TB, it seems reasonable that serum TB is negatively correlated with cardiovascular diseases. Accordingly, previous studies have shown that elevated TB positively impacted adverse cardiovascular events among patients with cardiovascular diseases 27, 28. In 2862 normal individuals who underwent coronary computed tomography, Kang et al. discovered that TB levels are negatively associated with the extent of coronary atherosclerosis and calcified plaques^29^. Similarly, Ozeki et al. examined the relationship between TB level and ankle-brachial index in 935 patients, which reported a significant negative correlation between TB level and peripheral artery disease 30. In addition to predicting adverse clinical outcomes, TB was also reported to have prognostic value in predicting contrast-induced nephropathy. A retrospective study enrolled 544 angina, or AMI patients who underwent coronary intervention procedures, showed that lower TB concentrations were positively associated with a higher risk of developing contrast-induced nephropathy 31. When it comes to emergencies, such as MI, the protective effect of TB might be altered. Recent studies have shown that higher TB was associated with higher in-hospital mortality among ST-segment elevation myocardial infarction (STEMI) patients 16, 32-34. A large meta-analysis by Shen et al. combined results obtained from six studies involving 14,554 AMI patients, showing that higher serum TB was positively associated with higher MACE among AMI patients 18. An earlier study reported that high TB was an independent predictor of no-reflow and in-hospital adverse outcomes in STEMI patients who received the primary percutaneous coronary intervention (PCI)^35^. Another study also found that higher TB was similarly linked to an elevated risk of MACE in patients with acute coronary syndrome 36. In addition, a previous study found that TB was positively associated with high SYNTAX scores in STEMI patients 37.

To date, several studies have investigated the association between TB and clinical outcomes in AMI. To the best of our knowledge, there are no specific clinical studies regarding the prognostic value of TB in MINOCA patients, a unique type of MI that requires specific attention. In the present study, for the first time, we showed that the high TB was significantly correlated with an increased risk of MACE, which was in line with previous AMI studies. The reasons might be listed as follows; firstly, a higher baseline TB level indicates more significant clinical abnormalities; we observed that the LVEF values of patients in the high TB group were significantly lower than those in the low TB group, which reflects the poorer cardiac function of patients in the high TB group to some extent. Secondly, elevated bilirubin levels might result from insufficient perfusion of the liver or hepatic congestion induced by cardiac failure. In addition, acute stress reaction might also be related to this positive correlation. Under circumstances of MI or pressure overload, heme oxygenase-1, which decomposes heme into bilirubin under oxidative stress, was found to be significantly up-regulated in animal models 38. Thus, a higher TB level could also reflect more severe myocardial damage in the acute clinical conditions, explaining the positive association between TB and MINOCA patients' prognosis.

Currently, there is no exact TB cut-off value for predicting adverse events in AMI patients. A previous study by Gul et al. stratified STEMI patients who underwent PCI into two groups based on a cut-off value of 0.9 md/dl for TB and found that in-hospital mortality was significantly higher in the high TB group 16. Moreover, another study stratified 1111 STEMI patients based on the cut-off value of 0.79 mg/dl for TB and found that the high TB group had a higher rate of adverse events 39. However, in the present study, we found that initial serum TB > 0.9mg/dl is an independent predictor of unfavorable outcomes in MINOCA patients, even after adjusting for confounding factors known to be associated with MACE among MINOCA patients. The ROC analysis indicated that TB>0.9mg/dl has importance in predicting long-term MACE among MINOCA patients.

Taken together, our findings suggested that serum TB, which is a cheap and easy-to-measure biomarker, could be effectively used for risk stratification of MINOCA patients. As a regular biochemical measure and a potent prognostic indicator for MINOCA patients, serum TB is readily available to clinicians.

Several limitations need to be considered. Firstly, this was a retrospective study, so the probability of selection bias and unmeasured variables cannot be ruled out. Secondly, this was a limited sample size single-center study. Thirdly, the clinical application value of serum TB was not matched to other inflammatory markers such as C reactive protein or myeloperoxidase. In addition, we did not record the dynamic changes in serum TB levels during the hospitalization and follow-up, as well as the lack of serum TB concentrations in the control group, which might also be clinically significant. More prospective studies with a larger sample size are required, however, to confirm the current study's findings.

## Conclusion

High initial serum TB (>0.9mg/dl) is a robust predictor of adverse clinical outcomes among MINOCA patients. In clinical practice, assessment of TB may aid in identifying high-risk MINOCA patients.

## Figures and Tables

**Table 1:** Baseline characteristics of the two groups stratified by serum total bilirubin

Variable	Total n=273	Low TB group n=205	High TB group n=68	*P* value
Age, n (%)	63.30±13.27	62.94±13.63	64.40 ±12.16	0.440
Male, n (%)	135 (49.5)	98 (47.8)	37 (54.4)	0.345
BMI, kg/m^2^	24.09±3.77	24.02±3.75	24.31±3.89	0.473
History of disease				
Hypertension, n (%)	139 (50.9)	105 (51.2)	34 (50.0)	0.862
Diabetes, n (%)	46 (16.8)	36 (17.6)	10 (14.7)	0.586
Dyslipidemia, n (%)	41 (15.0)	34 (16.6)	7 (10.3)	0.208
Atrial fibrillation, n (%)	23 (8.6)	18 (8.8)	5 (7.8)	0.809
Heart failure, n (%)	5 (1.8)	4 (2.0)	1 (1.5)	1.000
Admission characteristics				
STEMI, n (%)	109 (39.9)	75 (36.6)	34 (50.0)	0.050
NSTEMI, n (%)	164 (60.1)	130 (63.4)	34 (50.0)	0.050
LVEF (%)	55.25±10.98	56.31±10.25	52.08±12.47	0.003
SBP (mmHg)	142.06±23.06	140.91±23.16	145.51±22.57	0.414
DBP (mmHg)	80.97±13.15	80.35±13.14	82.82±13.11	0.739
Heart rate, beats per minute	81.44±17.47	80.90±17.80	83.06±16.45	0.527
Laboratory test				
TB (mg/dl)	0.72±0.33	0.57±0.18	1.17±0.25	0.032
ALT (U/L)	31.16±30.46	30.28±33.07	33.80±20.68	0.410
AST (U/L)	49.95±70.08	46.76±61.83	59.59±90.40	0.191
GGT (U/L)	41.06±54.33	40.15±56.90	43.95±45.53	0.670
Blood urea nitrogen (mmol/L)	6.24±2.90	6.15±3.00	6.51±2.59	0.549
Creatinine (mmol/L)	80.9±43.29	80.34±43.26	82.6±43.66	0.540
Total cholesterol (mmol/L)	4.27±1.07	4.30±1.06	4.18±1.07	0.416
Triglycerides (mmol/L)	1.52±1.09	1.62±1.18	1.23±0.67	0.029
cTnT (ng/mL)	0.46±1.06	0.44±0.93	0.5±1.38	0.217
NT-proBNP (pg/mL)	2046.74±4620.11	1906.75±4672.62	2468.78±4465.17	0.428

**Abbreviations:** BMI: body mass index; STEMI: ST-segment elevation myocardial infarction; NSTEMI: non-ST-segment elevation myocardial infarction; LVEF: left ventricular ejection fraction; SBP: systolic blood pressure; DBP: diastolic blood pressure; TB: total bilirubin; ALT: alanine aminotransferase; AST: aspartate aminotransferase; GGT: γ-glutamyltransferase; NT-proBNP: N-terminal pro-B-type natriuretic peptide.

**Table 2:** Accumulated major adverse cardiovascular events during the study endpoints

	Low TB group N=205	High TB group N=68	*P* value
**MACE**	35(17.1%)	21(30.9%)	0.015
Cardiovascular death	13 (6.3%)	4 (5.9%)	1.000
Non-fatal myocardial infraction	0	2 (2.9%)	0.061
Heart failure	2 (1.0%)	1 (1.5%)	0.569
Angina rehospitalization	20 (9.8%)	14 (20.6%)	0.019

**Abbreviations:** MACE: major adverse cardiovascular events.

**Table 3:** The association between serum total bilirubin and clinical outcomes.

	Unadjusted			Model 1				Model 2	
Group	HR (95% CI)	*P* value		HR (95% CI)	*P* value			HR (95% CI)	*P* value
TB, per 1SD increase	1.45 (0.15-0.82)	0.001		1.40 (0.11-0.76)	0.004			1.43 (0.07-0.92)	0.016
Low TB group	Reference			Reference				Reference	
High TB group	1.90 (1.12-3.22)	0.018		1.83 (1.08-3.12)	0.026			2.04 (1.05-3.94)	0.034
									

Model 1 included sex and age. Model 2 included age, sex, BMI, diabetes, hypertension, hyperlipemia, atrial fibrillation, heart failure, left ventricular ejection fraction.
